# The Nutritional Efficacy of *Chlorella* Supplementation Depends on the Individual Gut Environment: A Randomised Control Study

**DOI:** 10.3389/fnut.2021.648073

**Published:** 2021-05-31

**Authors:** Yuichiro Nishimoto, Tatsuhiro Nomaguchi, Yuka Mori, Masaki Ito, Yuya Nakamura, Masaki Fujishima, Shinnosuke Murakami, Takuji Yamada, Shinji Fukuda

**Affiliations:** ^1^Metabologenomics, Inc., Tsuruoka, Japan; ^2^Sun Chlorella Corp., Kyoko, Japan; ^3^Institute for Advanced Biosciences, Keio University, Tsuruoka, Japan; ^4^Department of Life Science and Technology, Tokyo Institute of Technology, Tokyo, Japan; ^5^Intestinal Microbiota Project, Kanagawa Institute of Industrial Science and Technology, Kawasaki, Japan; ^6^Transborder Medical Research Center, University of Tsukuba, Tsukuba, Japan

**Keywords:** prebiotics (sources:MeSH), gut microbiome, gut metabolome, dietary fibre, *Chlorella*

## Abstract

Recent studies have accumulated evidence that the intestinal environment is strongly correlated with host diet, which influences host health. A number of dietary products whose mechanisms of influence operate via the gut microbiota have been revealed, but they are still limited. Here, we investigated the dietary influence of *Chlorella*, a green alga commercially available as a dietary supplement. A randomised, double-blind, placebo-controlled crossover trial including 40 Japanese participants with constipation was performed. In this study, the primary outcome and secondary outcome were set as defecation frequency and blood folate level, respectively. In both outcomes, no significant differences were detected compared to the control intake. Therefore, we analysed the gut microbiome, gut metabolome, and blood parameters in an integrated manner as an exploratory analysis. We revealed that the consumption of *Chlorella* increased the level of several dicarboxylic acids in faeces. Furthermore, the analysis showed that individuals with low concentrations of faecal propionate showed an increase in propionate concentration upon *Chlorella* intake. In addition, increasing blood folate levels were negatively correlated with defecation frequency at baseline. Our study suggested that the effect of *Chlorella* consumption varies among individuals depending on their intestinal environment, which illustrates the importance of stratified dietary management based on the intestinal environment in individuals.

## Introduction

*Chlorella* is a genus that belongs to the class Chlorophyceae and consists of mostly green algae living in freshwater. *Chlorella* species are protein rich and are currently well known as a dietary supplement that is commercially available and consumed by various populations. Recent studies have reported that *Chlorella* has various beneficial effects, such as anti-inflammatory (1) and anti-allergic (2) effects, lipid metabolism improvement (3), and improvement of some markers of several cardiovascular risk factors (4). *Chlorella* species also include dietary fibre. It has been reported that dietary fibre has effects such as lowering blood glucose levels, lowering cholesterol levels and suppressing intestinal inflammation (5–7). However, dietary fibre has a variety of sugar compositions, and some of its positive effects on human health depend on this composition (8). It is important to investigate the effect of each food that contains dietary fibre. In addition, the effect of dietary fibre sometimes depends on the host gut microbiota, as the gut microbiota is involved in its mechanism of action. For instance, improvement in glucose metabolism by barley consumption is associated with increased abundance of the intestinal bacterial genus *Prevotella* (7). The improvement of glucose metabolism by succinate, which is produced from dietary fibre by *Prevotella*, has been proposed as the mechanism of action (9). Rat studies have reported that *Chlorella* intake improved blood lipid profiles, which was correlated with gut microbiota alteration (3, 10). However, the gut microbiota is known to differ between rodents and humans (11); thus, the effect of *Chlorella* intake into the intestinal environment in humans could differ from that in rats. A previous study reported that there are inter-individual differences in the response to *Chlorella* intake (12); however, while the study focused on genetic differences among consumers, individual differences in the intestinal environment were not considered. The intestinal environment differs among human individuals (13). In this interventional study, we recruited participants with a tendency for constipation to evaluate their responses to *Chlorella* intake. In particular, this study focused on their bowel movement, blood parameters and intestinal environment analysed by a metabologenomics approach which is an integrated approach of 16S rRNA gene-based microbiome and mass spectrometry-based metabolome analyses.

## Result

### *Chlorella* Intake Affected the Abundances of Dicarboxylic Acid Metabolites in Faeces

**Figure 1:**
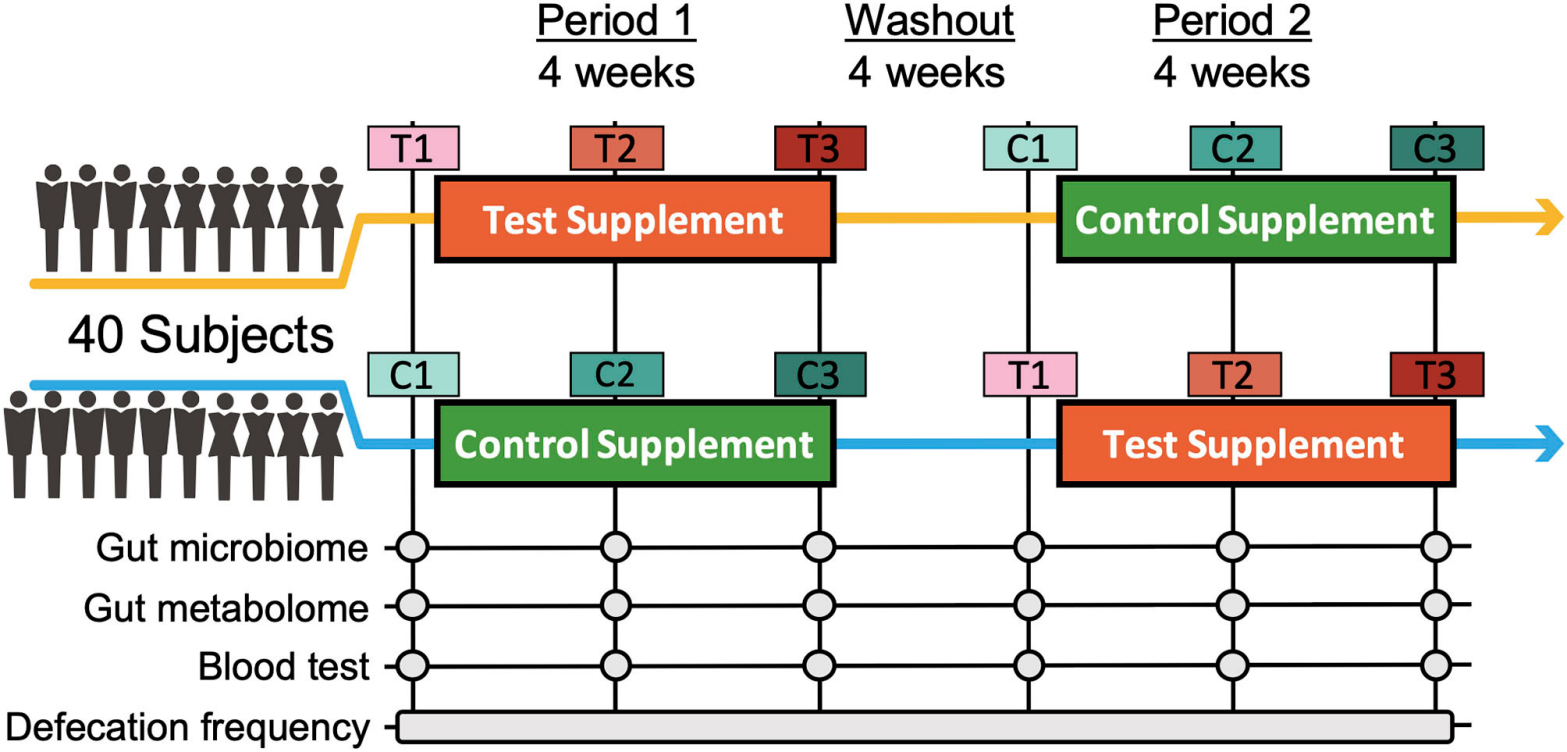
Timeline of the double-blind randomized crossover trial. Two 4-week dietary treatments were set in succession. The dietary intervention periods were interspaced by a 4-week washout period. Blood and faecal samples were collected beforehand and at week 2 and 4 of each intervention period. C and T indicate the control and test supplement intervention periods, respectively, and number 1-3 indicates the sampling timepoint in the period.

To assess the effects of *Chlorella* intake, a randomised, controlled crossover trial with two 4-week dietary intervention periods separated by a 4-week washout period was performed (Figure 1). A total of 40 Japanese participants with constipation tendencies passed the inclusion criteria, and all of the participants completed the two dietary intervention periods. Participants in two randomised blocks had similar clinical characteristics regarding the primary and secondary outcomes (defecation frequency and blood folate level) before the dietary intervention (Supplementary Table 1).

For the primary and secondary outcomes, changes in defecation frequency and blood folate level, which are hypothesised to both be improved by *Chlorella* intake (12, 14), were analysed. Fasting blood samples were taken before and during each intervention period, and folate levels were biochemically analysed. Participants were asked to record their defecation frequency during the intervention periods. The defecation frequencies during days 14–28 in the control intervention period and the *Chlorella* intervention period were compared; however, no significant differences were observed in either defecation frequency or blood folate levels (Table 1).

**Table 1:** *Chlorella* intake did not significantly increase defecation frequency or blood folate levels.

	**Effect size^**a**^**	***p*-value^**b**^**	**C1^**c**^**	**T1^**c**^**	**C3^**c**^**	**T3^**c**^**
Defecation frequency^d^ (/week)	0.101 (−0.426 to 0.628)	0.702	3.95 ± 1.05	4.15 ± 1.42	4.98 ± 1.36	5.30 ± 1.99
Blood folate level (ng/ml)	0.208 (−0.604 to 1.020)	0.607	9.21 ± 4.29	8.48 ± 3.39	8.10 ± 3.76	7.84 ± 2.95

a *Values are given as the mean and 95% confidence interval*.

b *Values indicate paired t-test result*.

c *Values are given as mean ± standard deviation (S.D.)*.

d *Values represent the mean defecation frequency per week, which was recorded for 2 weeks; C1 and T1 were recorded before the start of the placebo and test intervention periods, respectively. C3 and T3 were recorded during the 3rd and 4th weeks of each intervention period*.

**Figure 2:**
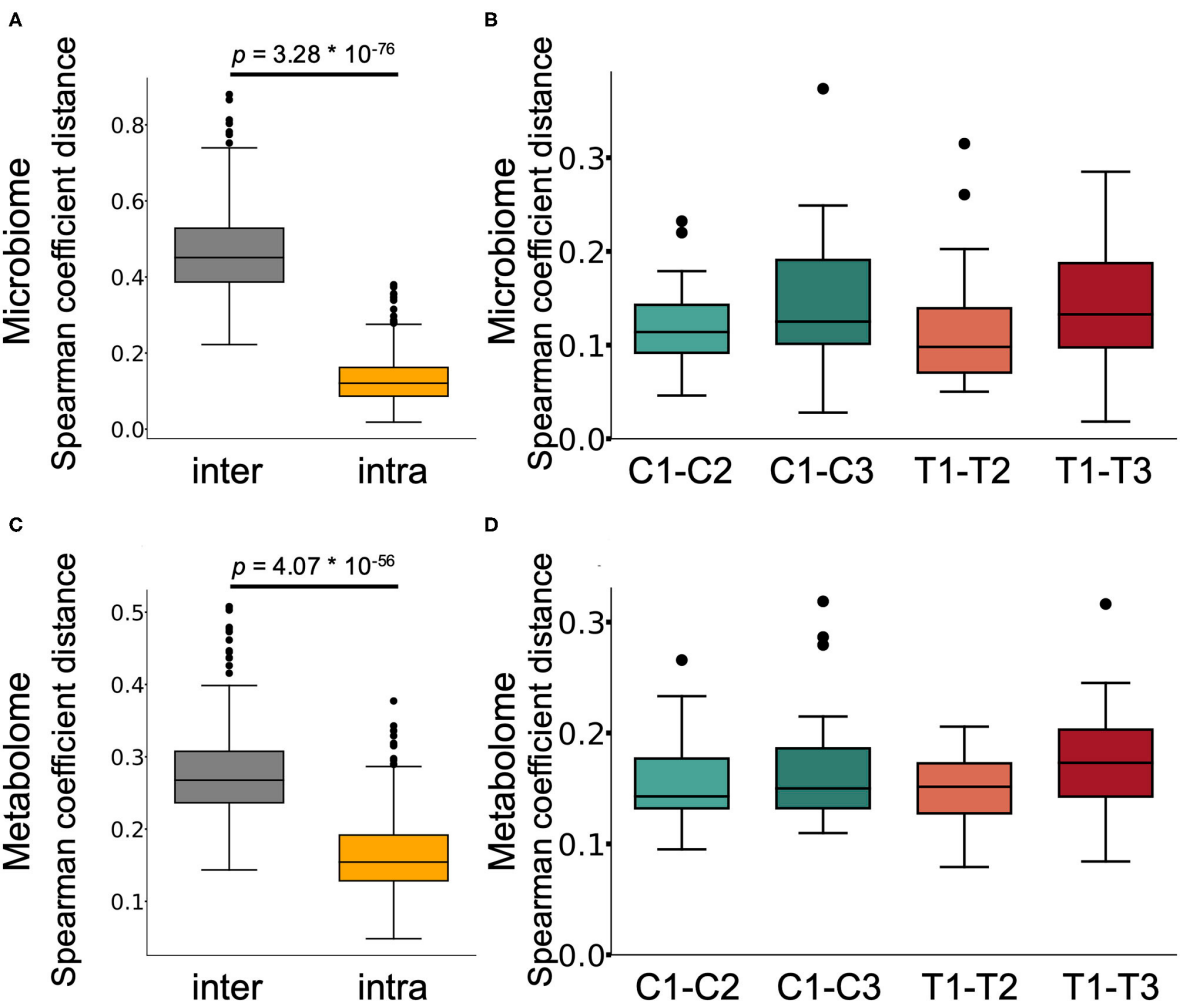
The influence of *Chlorella* consumption on the gut microbiome and metabolome profiles was not larger than that of control supplement consumption. **(A,C)** Box plot representing the distribution of Spearman coefficient distance for the gut microbiome **(A)** and metabolome profiles **(C)** among samples from different subjects at the first timepoint (inter) and the distance between samples from the same subject (intra). **(B,D)** The boxplots show the microbiome profile distance **(B)** and metabolome profile distance **(D)** within an individual between the labelled timepoints. A significant difference test was performed during the same intake period (T1–T2 and C1–C2, or T1–T3 and C1–C3), but no significant difference was detected (Wilcoxon signed-rank test).

We next performed 16S rRNA-based microbiome analysis and capillary electrophoresis time-of-flight mass spectrometry (CE-TOFMS)-based metabolome analysis. In this study, 20 randomly selected subject samples were analysed. Faecal samples were obtained prior to intervention (C1 and T1 in Figure 1. C and T indicate the control supplement and test supplement intervention periods, respectively, and 1 indicates the first sampling timepoint during the intervention) and at ~14 days (C2 and T2) and 28 days (C3 and T3) into each intervention period (Figure 1). Multivariate analysis based on the Spearman rank correlation coefficient showed that the inter-individual distance in microbiome and metabolome profiles was significantly higher than the intra-individual distance (Figures 2A,C; *p* = 3.28 × 10^−76^ and 4.07 × 10^−56^ when using the microbiome and metabolome profiles, respectively; Wilcoxon rank sum test). This indicated that inter-individual variation in microbiome and metabolome profiles is more prominent than the effect caused by the intervention. Therefore, to evaluate the effect of *Chlorella* intake on the microbiome and metabolome profiles, it is necessary to compare variations within the individual. We compared intra-individual variation before and after placebo intake (C1 and C2, and C1 and C3) and intra-individual variation before and after *Chlorella* intake (T1 and T2, and T1 and T3), but no significant difference was detected (Figures 2B,D). These results indicated that *Chlorella* intake had no significant effect on the general profile of the microbiome and the metabolome.

**Figure 3:**
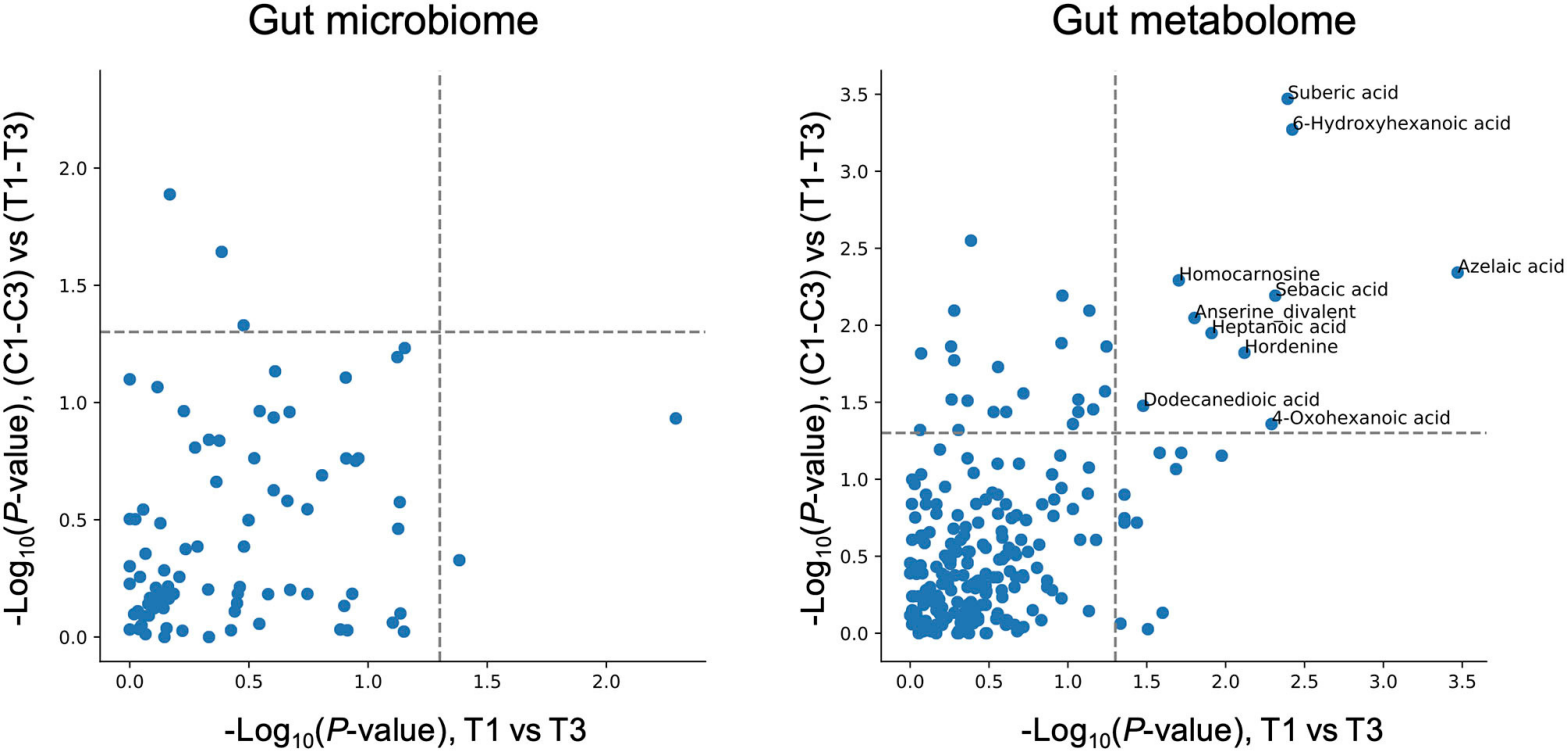
*Chlorella* intake significantly alters some intestinal metabolite levels. The *X*-axis indicates the logarithmic *P*-value of the Wilcoxon signed-rank test for each bacterial taxon/metabolite between T1 and T3. The *Y*-axis indicates the logarithmic *P*-value of the Wilcoxon signed-rank test for each bacterial taxon/metabolite between T1–T3 and C1–C3 (T1–T3 means differences between T1 and T3 within each subject treated as one group). Bacterial taxon/metabolite names are labelled if a significant difference was detected in both statistical tests.

While *Chlorella* intake had no significant effect on the entire microbiome and metabolome profiles, some of the specific intestinal bacteria or metabolites may have been affected. To assess the effect of intervention on each bacterium and metabolite, the relative abundance of each bacterium and scaled peak areas of each metabolite, which represent the amount of each metabolite in faeces, were analysed. During the analysis, we performed the Wilcoxon signed-rank test with two sets of pairs: comparison between T1 and T3 for comparison before and after *Chlorella* intake and comparison between T1–T3 and C1–C3 for comparison of the effect caused by control supplement and *Chlorella* intake (T1–T3 and C1–C3 represent the difference in values between the 3rd and 1st timepoints of the test supplement and the control supplement intervention periods, respectively, in each subject). Although significant differences in bacterial abundance were detected in either one comparison or the other, 10 metabolites showed significant differences in both comparisons (Figure 3). The functions of these metabolites were analysed using the KEGG BRITE database, and the results revealed that half of these metabolites were dicarboxylic acids (Table 2). Three metabolites, namely, azelaic acid, suberic acid, and 6-hydroxyhexanoic acid, remained significant after false discovery rate (FDR) adjustment by the Benjamini-Hochberg (BH) approach. Azelaic acid remained significant after FDR adjustment in the comparison between T1 and T3; suberic acid and 6-hydroxyhexanoic acid remained significant after FDR adjustment in the comparison between T1–T3 and C1–C3. *Chlorella* itself may contain these three metabolites (azelaic acid, suberic acid, and 6-hydroxyhexanoic acid). Therefore, CE-TOFMS metabolic profiling was performed to measure the nutritional contents in *Chlorella*. However, these three metabolites were not present in *Chlorella* (Supplementary Table 2).

**Table 2:** *Chlorella* intake increases dicarboxylic acid levels in faeces.

	***p*-value^a^**	***p*-value^b^**	**C1^c^**	**C3^c^**	**T1^c^**	**T3^c^**	**Brite1**	**Brite2**	**Brite3**
**Azelaic acid**	0.0003	0.0045	0.0067 (0.0049)	0.0062 (0.0046)	0.0051 (0.0027)	0.0078 (0.0049)	Fatty acyls	Fatty Acids and Conjugates	Dicarboxylic acids
**6-Hydroxyhexanoic acid**	0.0038	0.0005	0.0058 (0.0090)	0.0040 (0.0068)	0.0050 (0.0068)	0.0084 (0.0094)	Fatty acyls	Fatty Acids and Conjugates	Hydroxy fatty acids
**Suberic acid**	0.0040	0.0003	0.0031 (0.0016)	0.0024 (0.0011)	0.0024 (0.0007)	0.0036 (0.0018)	Organic acids	Carboxylic acids	Dicarboxylic acids
							Fatty acyls	Fatty Acids and Conjugates	Dicarboxylic acids
**Sebacic acid**	0.0048	0.0064	0.0015 (0.0009)	0.0012 (0.0010)	0.0010 (0.0008)	0.0019 (0.0013)	Fatty acyls	Fatty Acids and Conjugates	Dicarboxylic acids
**4-Oxohexanoic acid**	0.0051	0.0438	0.0013 (0.0007)	0.0013 (0.0005)	0.0011 (0.0007)	0.0016 (0.0006)	N.A.	N.A.	N.A.
**Hordenine**	0.0076	0.0151	0.0002 (0.0002)	0.0002 (0.0002)	0.0002 (0.0002)	0.0001 (0.0002)	Alkaloids	Alkaloids derived from tyrosine	Tyramine derivatives
**Heptanoic acid**	0.0123	0.0112	0.0052 (0.0122)	0.0042 (0.0083)	0.0046 (0.0109)	0.0066 (0.0108)	Organic acids	Carboxylic acids	Monocarboxylic acids
							Fatty acyls	Fatty Acids and Conjugates	Straight chain fatty acids
**Anserine_divalent**	0.0158	0.009	0.0014 (0.0020)	0.0016 (0.0016)	0.0020 (0.0020)	0.0009 (0.0009)	N.A.	N.A.	N.A.
**Homocarnosine**	0.0198	0.0051	0.0003 (0.0003)	0.0004 (0.0004)	0.0004 (0.0003)	0.0002 (0.0003)	N.A.	N.A.	N.A.
**Dodecanedioic acid**	0.0333	0.0333	0.0105 (0.0169)	0.0064 (0.0065)	0.0049 (0.0039)	0.0140 (0.0233)	Fatty acyls	Fatty Acids and Conjugates	Dicarboxylic acids

*Classification of significantly enriched faecal metabolites upon Chlorella intake based on the KEGG BRITE database (p < 0.05 in both comparisons between C1–C3 and T1–T3 and between T1 and T3)*.

a *Values indicate Wilcoxon signed-rank test result between T1 and T3*.

b *Values indicate Wilcoxon signed-rank test result between C1–C3 and T1–T3*.

c *Values are given as the mean (S.D.)*.

### Correlation Analysis Revealed That the Effect of *Chlorella* Intake Depends on the Individual Intestinal Environment

**Figure 4:**
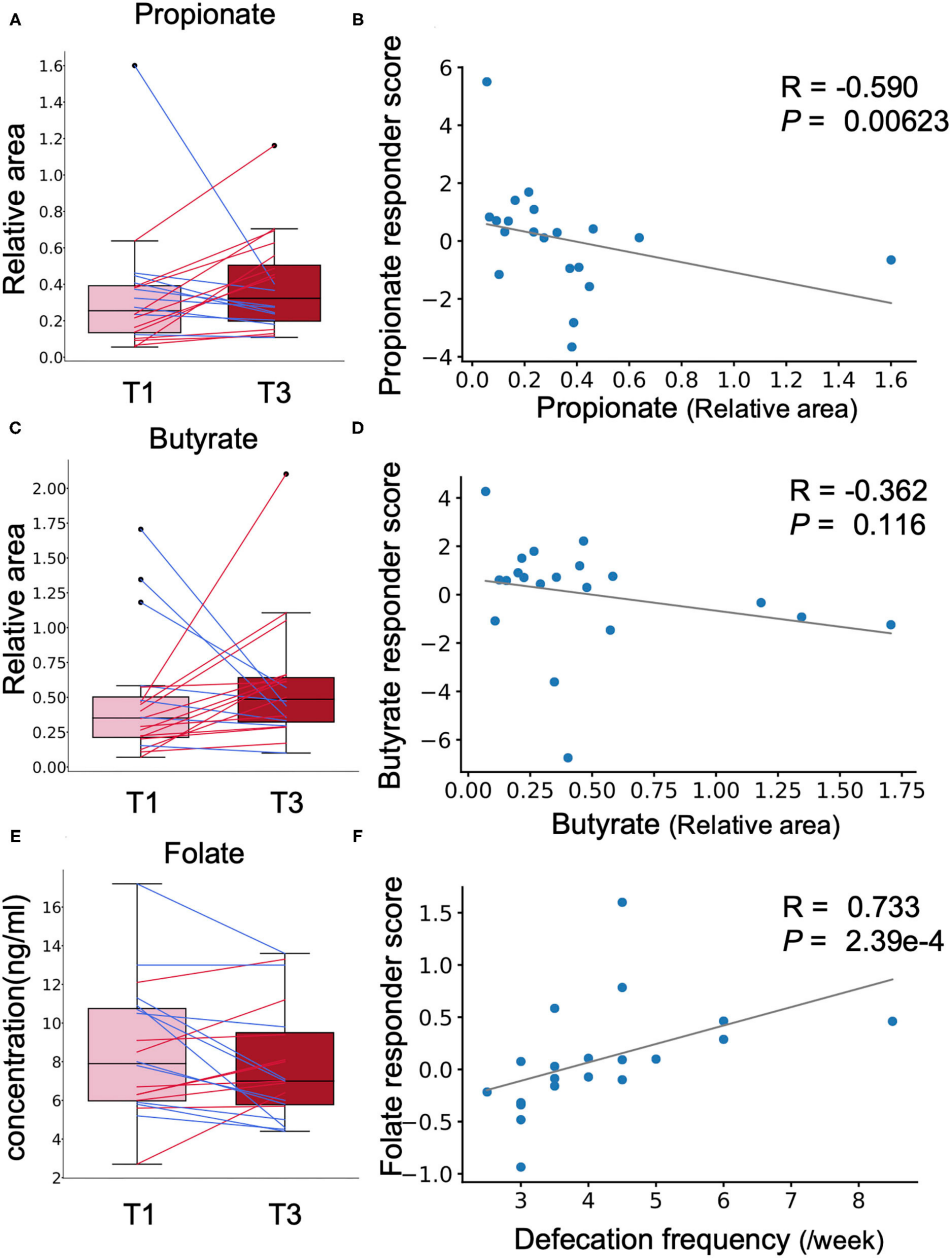
The effect of *Chlorella* intake depends on intestinal environmental factors at baseline. **(A,C,E)** Box plots representing the distribution of faecal propionate **(A)**, butyrate **(C)**, and blood folate **(E)** concentrations. The relative area shows the peak area ratio of samples compared to the internal standard, which represents the concentration of the corresponding metabolite. No significant difference was detected (Wilcoxon signed-rank test). **(B,D,F)** The *X*-axis indicates the value at baseline. The *Y*-axis indicates the responder score of each metabolite/blood test item. Correlation was determined using the Spearman coefficient. The grey line shows the approximated curve.

*Chlorella* contains dietary fibre such as β-glucan that is metabolised to short-chain fatty acids (SCFAs) such as propionate and butyrate, which are known to have beneficial effects on host health, by the gut microbiota (15). However, a simple comparison showed no significant difference in the faecal abundances of propionate and butyrate before and after *Chlorella* intake in this study (Figures 4A,C). A previous study has reported that the effects of probiotics on SCFAs in faeces differ between individuals depending on their SCFA levels at baseline (16). We analysed the correlation between the effect size of *Chlorella* intake, which will be referred as the responder score (see section Methods for details), and the baseline faeces propionate and butyrate levels of each individual. The propionate responder score showed a negative correlation with the propionate levels at baseline (Figures 4A,B; Spearman *r* = −0.589; *p* = 0.00623). This indicates that subjects with low propionate levels in faeces before *Chlorella* consumption showed an increase in propionate after *Chlorella* intake. Butyrate also exhibited a similar tendency, but the correlation was not significant (Figures 4C,D; Spearman *r* = −0.362; *p* = 0.116).

SCFAs are major faecal metabolites that are produced via the metabolism of dietary fibre by the gut microbiota. Therefore, to evaluate the bacteria contributing to the response, a correlation analysis was performed on the propionate and butyrate responder scores and the abundance of each intestinal bacterial taxon at baseline. The abundances of the two bacteria (*Ruminiclostridium* 9 and *Butyricimonas*) were significantly correlated with both propionate and butyrate responder scores (Supplementary Table 3). Bacteria that were correlated significantly with propionate or butyrate responder scores are listed in Supplementary Table 3.

Next, we performed the same analysis with primary and secondary outcomes (defecation frequency and blood folate level) to evaluate whether the effect on these outcomes was also dependent on prior individual intestinal environments. To evaluate whether the effects on defecation frequency and blood folate level were dependent on the intestinal environment, correlation analysis was performed for defecation frequency responder score, blood folate responder score, and intestinal environment factors, including intestinal bacteria and metabolite abundances and defecation frequency at baseline. A strong positive correlation was found between blood folate responder score and defecation frequency at baseline (Figures 4E,F; Spearman *r* = 0.733; *p* = 2.39 × 10^−4^). Other intestinal environmental factors that were significantly correlated with defecation frequency or blood folate responder score are listed in Supplementary Table 4.

## Discussion

In our study, no significant differences in defecation frequency or blood folate level were observed between timepoints C3 and T3. One possible reason was the placebo effect caused by the intervention. When we compared the defecation frequency between T1 and T3 with a statistical pairwise test, a significant difference was detected. However, there was also a significant difference observed between C1 and C3, which correspond to times before and after control supplement intake, respectively. This suggests that placebo effects appeared during both intake periods; thus, the effect caused by *Chlorella* intake became difficult to detect when C1–C3 and T1–T3 were compared.

Multivariate analysis showed that *Chlorella* intake had no significant effect on the general profiles of either the gut microbiome or metabolome. This is the same tendency as that found in our previous research (17). While the general microbiome and metabolome profiles were not significantly affected, there were several metabolites that showed significant changes in abundance after *Chlorella* intervention. Azelaic acid showed an especially clear effect, since it was detected as a significantly increased metabolite even after FDR adjustment (T1 and T3 comparison, *q* < 0.10). Azelaic acid is a commonly contained nutrient in grain such as barley. Azelaic acid can improve glucose tolerance in a mouse model (18). This effect is consistent with the result in a previous barley study showing that barley can also improve glucose tolerance (7). Previous studies with rodents also reported that *Chlorella* intake improves glucose tolerance (19, 20), possibly via an increase in intestinal azelaic acid. In this study, fasting blood glucose levels did not improve significantly. Tests showing glucose tolerance, such as oral glucose tolerance tests, are needed to clarify the effect of *Chlorella* consumption.

Dicarboxylic acids, which were significantly enriched metabolites in this study, were not reported to be present in *Chlorella*. Indeed, metabolome analysis revealed that azelaic acid and suberic acid were not detected in *Chlorella* (Supplementary Table 2). Therefore, the observed increase was likely derived from the metabolism of other molecules originally present in *Chlorella*. *Chlorella* and many algae are known to produce and accumulate various long-chain fatty acid molecules (21). Such long-chain fatty acids are metabolised to dicarboxylic acids by ω-oxidation, which was also shown in an *in vivo* study in which fatty acids were administered to animals or humans that then excreted high levels of dicarboxylic acids in their urine (22). These reports suggest that the fatty acids in *Chlorella* may be metabolised to dicarboxylic acids, the levels of which increase in the blood. Dicarboxylic acids have been reported to have beneficial effects, such as antiketogenic effects; therefore, the increase in dicarboxylic acids caused by *Chlorella* intake would provide additional benefits other than the improvement of glucose tolerance discussed previously (23).

The effect derived from food consumption varies among individuals, and recent studies have pointed out that the intestinal environment is an important factor determining individual dependency. For instance, barley-induced improvement in glucose metabolism is associated with an increase in the gut microbial *Prevotella*/*Bacteroides* ratio (7). Therefore, it is important to stratify the population into groups depending on their response to the intervention and to study those groups separately to understand the truly important elements responsible for the effect. In this study, we performed a correlation analysis with our defined responder scores and discovered that subjects with a low concentration of SCFAs, especially propionate, had increased faecal SCFA levels. Although there were differences in whether the test supplement was probiotic or prebiotic, previous studies have shown the same tendency as our *Chlorella* intervention study (16). Propionate has beneficial effects, such as anti-inflammatory activity (24) and body weight maintenance (25). Since our data suggested that individuals with low concentrations of propionate showed an increase in propionate concentration upon *Chlorella* intake, there is a possibility that *Chlorella* intake reduces the risk of inflammation and obesity in individuals with low concentrations of propionate. We also analysed features of the gut microbiota and metabolites in subjects with increased propionate levels (Supplementary Table 3). In particular, the abundances of *Ruminiclostridium* 9 and *Butyricimonas* showed positive and negative correlations, respectively, with butyrate and propionate responder scores. There is a possibility that gut microbes that have a positive correlation with propionate and butyrate responder scores may be related to the production of propionate and butyrate and that those with a negative correlation may inhibit propionate and butyrate production. The genus *Ruminiclostridium* includes species such as *Ruminiclostridium cellulolyticum*, which is known as a cellulolytic bacterium (26). Therefore, efficient *Chlorella*-derived cellulose degradation by *Ruminiclostridium* 9 may have been involved in the production of short-chain fatty acids.

When we focused on the parameters related to blood folate levels, we discovered that the defecation frequency was positively correlated with the folate responder score. A previous study has shown that individuals with constipation tendencies have a lower absorption rate of nutrients than healthy individuals (27). This study showed that folate is also associated with constipation tendency. Our results may indicate that the absorbance of *Chlorella*-derived folate depends on defecation frequency.

The following limitations should be considered for our study. First, we analysed multivariate data, which included abundances of hundreds of intestinal bacterial taxa and metabolites. FDR correction is necessary in statistical hypothetical tests. However, the FDR correction was strict, as many items were comprehensively observed. Therefore, we discuss the factor detected by the multiple comparison without FDR adjustment. Since the factors detected by multiple comparisons without FDR adjustment possibly include false positives, validation with animal models or humans is crucial to confirm the findings. Second, this study was human intervention research that aimed to evaluate the effects of *Chlorella* intervention and elucidate the mechanism. To clarify the detailed mechanism, further study with animal models and molecular biological experiments are necessary. Third, the human gut microbiota is influenced by the host diet, but there was no assessment of the background diet in the present study. Although the intake of prebiotics and probiotics, which are known to affect the gut environment, was restricted during the trial, the possible influence of diet on the effects of *Chlorella* should be assessed in future studies.

In conclusion, we showed that the consumption of *Chlorella* increases the levels of several faecal dicarboxylic acids, such as azelate, which may improve glucose tolerance. On the other hand, the blood folate level, which has been reported to be increased by *Chlorella* consumption, was increased in only specific individuals with high defecation frequency. The data suggest that *Chlorella* intake with simultaneous intervention for improving bowel movement may enhance the effect of *Chlorella* consumption. In addition to the blood folate level, we discovered that the faecal propionate concentration was also improved by *Chlorella* consumption in individuals with low concentrations of propionate. Our results suggested that the effects derived from *Chlorella* consumption differ by individual based on their intestinal environments prior to intake. Such inter-individual differences in the effect of supplementation indicate the importance of a stratified healthcare approach that involves estimating the optimal nutrition for each individual to maximise the effects of diet.

## Methods

### Ethics Approval

The human rights of the subjects who participated in this study were protected at all times, and the study observed the Helsinki Declaration and the Ethical Guidelines on Epidemiological Research in Japan referring to standards for clinical trials of drugs. This trial was conducted with the approval of the clinical trial ethics review committee of the Chiyoda Paramedical Care Clinic and reported to https://www.umin.ac.jp/ (Identifiers: UMIN000041648).

### Trial Design and Recruitment

In this study, a randomised double-blind placebo-controlled crossover trial in Japanese participants was performed for 3 months between December 2016 and April 2017 (Figure 1; Supplementary Figure 1; Supplementary Table 5). The sample size was estimated based on a previous study (14). Eight subjects were required per group (significance level was 5% and statistical power was 80%). In total, 80 participants were recruited in this study. The participants fulfilled the following criteria: women aged between 20 and 60 years old with constipation (defecation frequency of 3–5 times per week). The detailed inclusion criteria were as indicated: (1) females aged 20–60 years; (2) defecation frequency of 3–5 times per week; (3)subjects who could visit the designated facility on the scheduled date; (4) understanding of the study procedures and agreement with participation in the study by written informed consent prior to the study. The detailed exclusion criteria were as indicated: (1) continuing to receive medical treatment; (2) constantly taking oral medicines, functional foods, and/or supplements that could affect the test results; (3) currently pregnant, lactating, or having the possibility of pregnancy; (4) donating over 200 mL of blood and/or blood components within the last 4 weeks to the current study; (5) donating over 400 mL of blood within the last 16 weeks to the current study; (6) over 800 mL of blood was collected when the amounts sampled within the last 12 months were added to the planned sampling amounts of this study; (7) participating in the other clinical test within 4 weeks before the start of the test; (8) having pain or bleeding during defecation; (9) having abdominal surgical operation within 6 months before the start of the test; (10) taking antibiotics within 6 months before the start of the test; (11) consuming an excessive amount of alcohol or smoking heavily; (12) having irregular dining habits and/or working midnight or irregular shifts; (13) planning a large change in lifestyle during the test period; (14) having a tendency for chronic diarrhoea; (15-a) having heart disease, liver disease, kidney disease, or diabetes (including the complication of other diseases); (15-b) having a medical history of cardiovascular diseases; (15-c) having an allergy to the test supplement; (16) determined ineligible by principal investigator or subinvestigator. Based on the inclusion/exclusion criteria, 40 subjects were selected for the main trial. Randomisation in this trial was performed using the block stratified randomisation method. Subjects were stratified by age and defecation frequency. Details regarding randomisation were as previously reported (13). The study included 4-week dietary intervention periods in which the subjects ingested the test supplement [3 g of *Chlorella pyrenoidosa* (Sun Chlorella A Tablets®, Sun Chlorella Corp., Kyoto, Japan) twice daily] and control supplement (3 g of digestible dextrin and pigment twice daily) in random order and separated by a 4-week washout period. Both the test supplement and control supplement were provided as tablets with similar appearances.

As this trial was designed as a crossover trial, all 40 volunteers consumed both the test and control supplements (Figure 1). The volunteers were divided into two groups (20 volunteers in each group) with stratified random sampling. Volunteers in each group consumed either test or control supplements during the first period and then consumed the other supplement during the second period. The order of consumption depended on the group to which the volunteers were assigned. At each timepoint, volunteers were asked to collect faecal samples three times: (1) before starting consumption, (2) 2 weeks after the start of consumption, and (3) 4 weeks after consumption. Timepoints were named [C1, C2, and C3] or [T1, T2, and T3], where C and T indicate the control and test supplements that the volunteers were consuming at that point. The collected faecal samples were frozen at −20°C until processing. Clinical blood tests were performed following 12 h of fasting at the same timepoint. In the clinical blood test, folate was measured by a chemiluminescence immunoassay as the key secondary outcome. As other outcomes from blood tests, total protein, albumin, aspartate aminotransferase, alanine transaminase, lactate dehydrogenase, total bilirubin, alkaline phosphatase, γ-glutamyl transpeptidase, creatine phosphate enzyme, urea nitrogen, creatinine, uric acid, sodium, chlorine, potassium, calcium, total cholesterol, low-density lipoprotein cholesterol, high-density lipoprotein cholesterol, neutral fat, glucose, white blood cells, red blood cells, haemoglobin, haematocrit, platelet, folate, vitamin B12, and homocysteine were measured. All subjects completed the trial, and microbiome and metabolome analyses were performed for 20 out of the 40 people for financial reasons. The primary outcome was defecation frequency, and the key secondary outcomes were faecal 16S rRNA metagenomic analysis, metabolome analysis and blood folate concentration.

### Faecal DNA and Metabolite Extraction

DNA extraction from faecal samples was performed as previously reported (28). After extraction, the V1–V2 variable region of the 16S rRNA gene was amplified using the bacterial universal primers 27F-mod (5′-AGRGTTTGATYMTGGCTCAG-3′) and 338R (5′-TGCTGCCTCCCGTAGGAGT-3′) with Tks Gflex DNA polymerase (TaKaRa Bio Inc., Japan) (29). The amplicon DNA was sequenced using MiSeq (Illumina, USA) according to the manufacturer's protocol. Extraction of metabolites from faecal samples was performed as previously reported (30). The obtained sequence data are available from DRA010607, and the metabolome data are available in Supplementary Table 6.

### *Chlorella* Metabolomic Analysis

CE-TOFMS metabolic profiling was performed to measure the nutritional contents in *Chlorella*. Fifty milligrammes of powdered *Chlorella* was vortexed with Milli-Q water for one minute and then agitated at 500 rpm and 37°C for 1 h. Samples were then centrifuged at 9,100 × *g* for 5 min. A total of 250 μL of supernatant was ultrafiltrated using an Ultrafree 5 kDa MWCO centrifugal philtre unit (Millipore) at 9,100 × *g* for 80 min. The internal standard was added to the filtrate and analysed using CE-TOFMS in both positive and negative modes. As a blank sample, a sample without *Chlorella* powder was also prepared and analysed.

### Bioinformatics and Statistical Analysis

For 16S rRNA gene analysis, QIIME2 (version 2019.10) was used (31). In the analytical pipeline, sequence data were processed by using the DADA2 pipeline for quality filtering and denoising (options: –p-trim-left-f 20 –p-trim-left-r 19 –p-trunc-len-f 240 –p-trunc-len-r 140) (32). The filtered output sequences were assigned to taxa by using the “qiime feature-classifier classify-sklearn” command with the default parameters. Silva SSU Ref Nr 99 (version 132) was used as a reference database for taxonomy assignment. In statistical analysis, the paired t-test and paired Cohen's d in the R package effsize were used for pairwise comparisons of primary and secondary outcomes. Other statistical analyses were performed with in-house Python scripts (version 3.7.3). For pairwise comparison of the relative abundances of intestinal bacterial taxa and the relative areas of intestinal metabolites, the Wilcoxon signed-rank test with Benjamini-Hochberg false discovery rate (FDR-BH) correction was used (scipy version 1.3.1 and statsmodels 0.10.1 were used for the Wilcoxon signed-rank test and FDR-BH correction, respectively). During the comparison, bacterial taxa with a mean relative abundance below 0.001 and metabolites not detected in 75% of samples were excluded.

### Defining the Responder Score With a Specific Response

In this study, the test supplement effect size was defined as the responder score and used to evaluate whether effects depended on individual basal characteristics. The response score was calculated with the following equation:

$$\begin{array}{r} Responder\;Score\;=\;\left(\left(T3\;-\;T1\right)\;-\;\left(C3\;-\;C1\right)\right)\;\\ /\;Average\left(C1,\;T1\right) \end{array}$$

## Data Availability Statement

The original contributions presented in the study are publicly available. This data can be found here: The obtained 16S rRNA gene sequence data are available in the DDBJ DRA (DRA accession number: DRA010607).

## Ethics Statement

The studies involving human participants were reviewed and approved by Chiyoda Paramedical Care Clinic. The patients/participants provided their written informed consent to participate in this study.

## Author Contributions

MF, TY, and SF: conceptualisation. YNi: data curation. YNi and YNa: formal analysis and visualisation. YM and MI: investigation. MF and SM: methodology. SF: project administration. YNi and TN: writing – original draft. YNi, TN, YM, MI, YNa, MF, SM, TY, and SF: writing – review and editing. All authors contributed to the article and approved the submitted version.

## Conflict of Interest

YNi, TN, YM, MI, YNa, and SM are employee of Metabologenomics, Inc.; TY and SF are founders of Metabologenomics, Inc., and MF is an employee of Sun Chlorella Corp.

## Supplementary Material

Supplementary Figure 1 Flow diagram of the phases of the randomized double-blind placebo-controlled crossover study.

Supplementary Table 1 Difference in primary/secondary outcome in baseline.

Supplementary Table 2 Relative area of metabolome in *chlorella*.

Supplementary Table 3 List of microbial taxa significantly correlated with SCFAs responder score.

Supplementary Table 4 List of microbial taxa significantly correlated with folate and defecation frequency responder score.

Supplementary Table 5 CONSORT checklist.

Supplementary Table 6 Relative area of metabolome in faeces.
