# Supplementary figures and images for: The Nutritional Efficacy of Chlorella Supplementation Depends on the Individual Gut Environment: A Randomised Control Study
Source: Front Nutr. 2021 May 31;8:648073. doi: 10.3389/fnut.2021.648073 (PMC8200412; doi:10.3389/fnut.2021.648073)

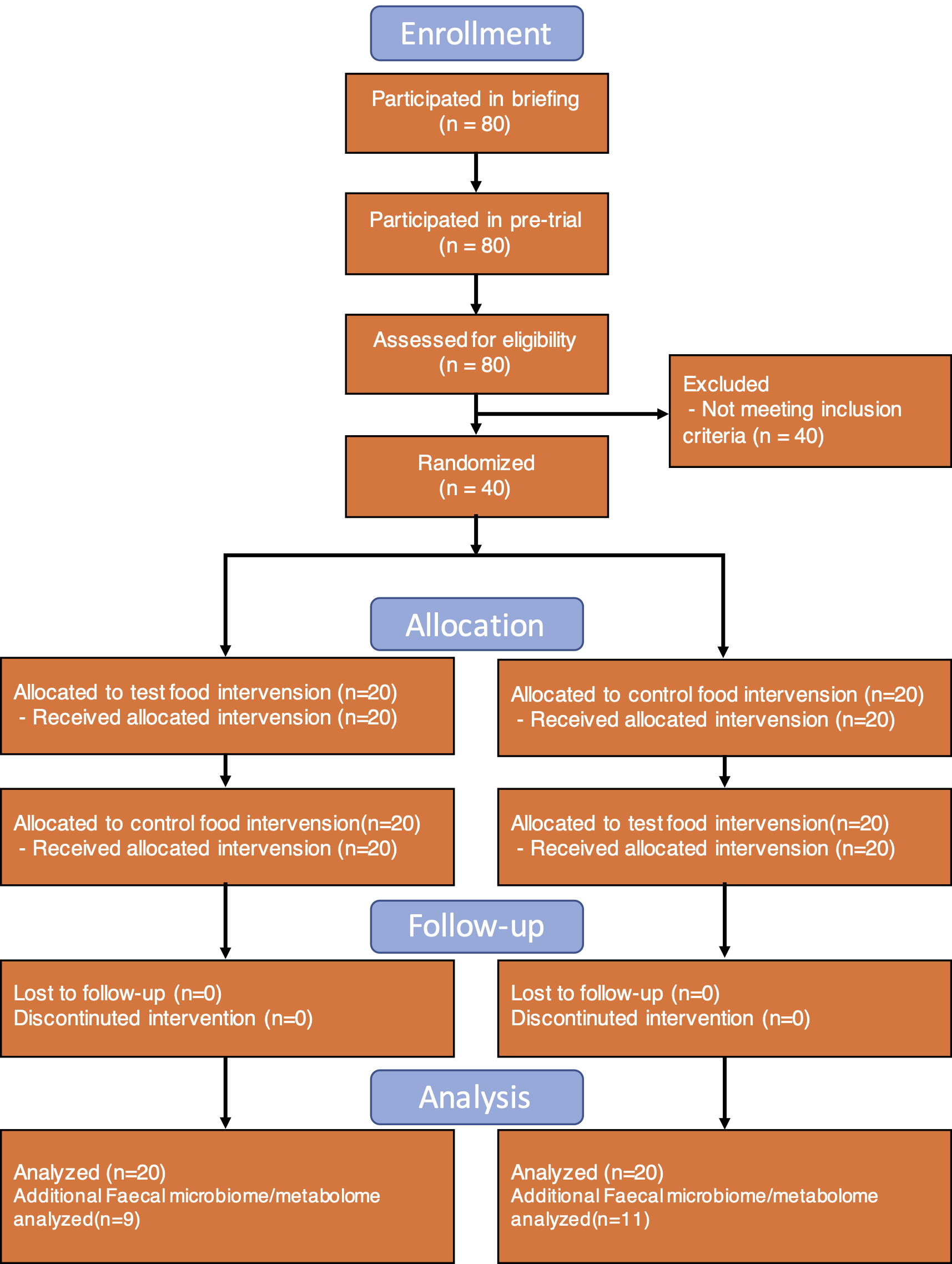

Supplement: Supplementary Figure 1 — Flow diagram of the phases of the randomized double-blind placebo-controlled crossover study. [file Image_1.TIFF]
